# Early Cytokine Release in Response to Live *Borrelia burgdorferi* Sensu Lato Spirochetes Is Largely Complement Independent

**DOI:** 10.1371/journal.pone.0108013

**Published:** 2014-09-29

**Authors:** Kerstin Sandholm, Anna J. Henningsson, Susanne Säve, Sven Bergström, Pia Forsberg, Nina Jonsson, Jan Ernerudh, Kristina N. Ekdahl

**Affiliations:** 1 Linnaeus University Centre for Biomaterials Chemistry, Linnaeus University, Kalmar, Sweden; 2 Department of Clinical Microbiology, Ryhov County Hospital, Jönköping, Sweden; 3 Department of Infection Medicine, and Department of Clinical and Experimental Medicine, Linköping University, Linköping, Sweden; 4 Department of Molecular Biology, University of Umeå, Umeå, Sweden; 5 Division of Clinical Immunology, Rudbeck Laboratory C5, University of Uppsala, Uppsala, Sweden; 6 Department of Clinical Immunology and Transfusion Medicine, and Department of Clinical and Experimental Medicine, Linköping University, Linköping, Sweden; Tulane University, United States of America

## Abstract

**Aim:**

Here we investigated the role of complement activation in phagocytosis and the release of cytokines and chemokines in response to two clinical isolates: *Borrelia afzelii* K78, which is resistant to complement-mediated lysis, and *Borrelia garinii* LU59, which is complement-sensitive.

**Methods:**

*Borrelia* spirochetes were incubated in hirudin plasma, or hirudin-anticoagulated whole blood. Complement activation was measured as the generation of C3a and sC5b-9. Binding of the complement components C3, factor H, C4, and C4BP to the bacterial surfaces was analyzed. The importance of complement activation on phagocytosis, and on the release of cytokines and chemokines, was investigated using inhibitors acting at different levels of the complement cascade.

**Results:**

1) *Borrelia garinii* LU59 induced significantly higher complement activation than did *Borrelia afzelii* K78. 2) *Borrelia afzelii* K78 recruited higher amounts of factor H resulting in significantly lower C3 binding. 3) Both *Borrelia* strains were efficiently phagocytized by granulocytes and monocytes, with substantial inhibition by complement blockade at the levels of C3 and C5. 4) The release of the pro-inflammatory cytokines and chemokines IL-1β, IL-6, TNF, CCL20, and CXCL8, together with the anti-inflammatory IL-10, were increased the most (by>10-fold after exposure to *Borrelia*). 5) Both strains induced a similar release of cytokines and chemokines, which in contrast to the phagocytosis, was almost totally unaffected by complement blockade.

**Conclusions:**

Our results show that complement activation plays an important role in the process of phagocytosis but not in the subsequent cytokine release in response to live *Borrelia* spirochetes.

## Introduction

Lyme borreliosis is an infectious disease that is predominantly found in the northern hemisphere and is caused by the spirochetes of *Borrelia burgdorferi* sensu lato (s.l.) [1]. The infection can affect the skin, joints, heart, and/or nervous system [2], [3]. At least three genospecies in the *B. burgdorferi* s.l. group have been identified as human pathogens [4] and have been shown to be associated with different clinical manifestations: *B. burgdorferi* sensu stricto (s.s.) is mainly associated with arthritis, *B. garinii* with neuroborreliosis, and *B. afzelii* with skin manifestations [1], [5], [6].

To survive in different hosts and tissues, it is crucial for the *Borrelia* spirochetes to overcome the human host's immune response. The innate immune system is the first line of defence that the spirochetes encounter when entering the body. These reactions are the key determinants of the magnitude and quality of the early immune response, and they subsequently initiate and form the adaptive immune response [7]. The complement system is part of innate immunity and consists of a complex network of plasma and membrane-associated proteins that are activated in a cascade-like manner. It can be activated through three different pathways: the classical, lectin, and alternative, all of which converge in a central step, the activation of C3 to generate C3b, which serves as both a ligand for immune adhesion and a subunit of the alternative pathway convertase. Complement activation induced by microbes leads to important defense mechanisms such as phagocytosis of the target and the formation of the membrane attack complex (MAC).

*Borrelia* species differ in their ability to survive in the presence of complement, and they are classified as serum-resistant or serum-sensitive, based on a quantification of the amount of MAC formed on the bacterial membrane *in vitro* [8]. *B. afzelii* is classified as serum-resistant and *B. burgdorferi* s.s. as moderately resistant, whereas *B. garinii* is sensitive to complement-mediated killing [9]–[12]. Studies have shown that resistance to complement is correlated with an ability of the isolates to recruit fluid-phase immune regulators, factor H (FH) and factor H-like-protein-1 (FHL-1), to the bacterial surface. This process promotes the inactivation and degradation of the alternative pathway C3 convertase and C3b, which in turn hinder downstream activation, leading to decreased formation of MAC and a higher rate of spirochete survival [10], [13], [14]. In addition, it has recently been reported that *B. burgdorferi* s.l. is capable of binding C4-binding protein (C4BP), a regulator of the classical and lectin pathways [15].

The complement regulators of the FH family bind to complement regulator-acquiring surface proteins (CRASPs) that are expressed on the surface of *B. burgdorferi* s.l., thereby reducing the alternative pathway of complement activation [16], [17]. Expression of the five known CRASPs has been identified on the serum-resistant *B*. *afzelii*; in contrast, *B*. *garinii*, which is serum-sensitive, expresses only one CRASP with weak or no binding to FHL-1 and FH [17], [18], indicating that resistance to complement is correlated with the expression of CRASPs.

In addition to its role in phagocytosis and microbial killing, complement activation is a major factor in early inflammation and an important contributor to the release of cytokines and chemokines such as the pro-inflammatory IL1-β, IL-6, TNF, and CCL20 (MIP-3α). These mediators are important for the recruitment of other components of the innate immune response as well as signaling with the adaptive immune system [19]. It is therefore reasonable to hypothesize that the early innate immune response, including complement activation, phagocytic capacity, and cytokine/chemokine release, is of great importance for the response to *B. burgdorferi* s.l.

The overall aim of this study was to characterize complement activation and its role in early immune activation in response to *Borrelia* spirochetes. *In vitro* models based on clinical isolates of *B. garinii* and *B. afzelii*, sensitive and resistant to complement activation, respectively, were used to investigate the importance of complement activation in phagocytosis and in the generation of cytokines and chemokines.

## Materials and Methods

### Heparin treatment of equipment

All equipment that came in contact with blood and plasma (tubes and pipette tips) was furnished with a heparin surface according to the manufacturer's recommendations (Corline AB, Uppsala, Sweden).

### Preparation of blood and plasma

Blood from healthy volunteers without (n = 8) or with (n = 1) specific anti-*Borrelia* antibodies was collected in 6-mL Vacutainer plastic tubes (BD Bioscience, Plymouth, UK) with the addition of the specific thrombin inhibitor hirudin (Refludan, Pharmion Ltd, Cambridge, UK), at a final concentration of 50 µg/mL blood. This study, using blood from healthy blood donors given their written consent, was performed with consent of the Ethical Committee of the University Hospital of Linköping, Sweden (#03-520). Plasma was collected by centrifugation at 3000 *g* for 20 min and stored at −80°C. For viability studies, aliquots of plasma were heat inactivated by incubation at 56°C for 30 min. For the phagocytosis experiments and cytokine release assays, blood was collected as described above and used within 30 min. Anti-*Borrelia* antibodies were measured in serum using the commercially available enzyme-linked immunosorbent assay (ELISA) kits Enzygnost Lyme link VlsE/IgG and Enzygnost Borreliosis IgM (DADE Behring, Marburg, Germany) on a BEP 2000 Advance System (Siemens Healthcare, Erlangen, Germany), according to the instructions from the manufacturer.

### Bacterial strains and growth conditions

The *Borrelia* strains used in this study were isolated in Sven Bergström's laboratory, Umeå University, Sweden: *B. garinii* LU59 from human cerebrospinal fluid (CSF) and *B. afzelii* K78 from a human skin biopsy. Bacteria were grown in Barbour-Stoenner-Kelly (BSK) II medium [30] supplemented with 7% rabbit serum (Sigma Aldrich, St. Louis, MO, USA) at 37°C until the density reached 10^8^–10^9^ cells/mL.

### Susceptibility of *B. garinii* LU59 and *B. afzelii* K78 to complement

In initial experiments, the susceptibility of both *Borrelia* strains to complement was investigated. Aliquots of *B. garinii* LU59 and *B. afzelii* K78 were incubated for 60 min at 37°C in serially diluted heat inactivated or native hirudin plasma, collected from a blood donor without *Borrelia*-specific antibodies. After incubation, the number of motile and non-motile spirochetes was counted in a phase contrast microscope [20], [21].

### Complement activation induced by *Borrelia*

Bacteria were washed with Dulbecco's PBS supplemented with 0.9 mM Ca^2+^ (PBSCa^2+^) by centrifugation at 4500 *g* for 5 min, then resuspended and diluted in PBSCa^2+^. Aliquots of 50 µL *B. garinii* LU59 and *B. afzelii* K78 spirochetes (10^9^/mL plasma) were incubated in 450 µL hirudin plasma (n = 8) without *Borrelia*-specific antibodies for 60 min at 37°C in 2-mL tubes. The reactions were stopped by the addition of ethylenediaminetetraacetic acid (EDTA, 10 mM final concentration; Sigma Aldrich) and centrifuged at 4500 *g* for 5 min. All incubations were performed in duplicate. Hirudin plasma incubated without spirochetes was used as a control. Complement activation was monitored as the generation of activation product C3a and sC5b-9 complexes, measured in the plasma by ELISA as described previously [22], [23]. Data are presented as the activation compared to a plasma control incubated in parallel, but without the presence of spirochetes.

### Quantification of C3/C4 fragments, FH, and C4BP by ELISA

*B. garinii* LU59 and *B. afzelii* K78 (10^9^/mL plasma) were incubated in hirudin plasma (n = 3) or PBSCa^2+^ at 37°C for 30 min and then centrifuged at 4500 *g* for 5 min. The pellets with spirochetes were washed three times with 500 µL PBSCa^2+^ and then resuspended in PBSCa^2+^ and transferred to new tubes. Biotinylated antibodies against human C3c (1.09 µg/mL), C4c (0.56 µg/mL), (Dako, Glostrup, Denmark), FH (1.13 µg/mL) and C4BP (1.44 ug/mL) (Binding Site, Birmingham, UK) were added, and the samples were incubated for 45 min at room temperature (RT). The samples were then centrifuged at 4500 *g* for 5 min and washed with wash buffer (PBS with 0.05% Tween 20; Sigma Aldrich) three times, then incubated with streptavidin-HRP (1∶500; GE Healthcare, Uppsala, Sweden), washed four times, and stained with o-phenylenediamine (OPD; Sigma Aldrich). The absorbance was measured at 492 nm.

### Visualization of C3 fragments and FH by immunostaining and Western blot

Spirochetes (10^9^/mL plasma) were incubated for 30 min in hirudin plasma or PBSCa^2+^ at 37°C. Complement activation was stopped by the addition of EDTA to a final concentration of 10 mM. Samples were centrifuged at 4500 *g* for 5 min, and the pellet containing the spirochetes was washed four times with PBSCa^2+^ and analyzed for C3 fragments and FH by immunostaining and Western blot. For immunostaining, the spirochetes were incubated for 30 min with polyclonal anti-bodies against human C3c, which also detects intact C3, C3b, and iC3b (200 µg/mL; Dako), FH (250 µg/mL; Binding Site), and *Borrelia* (280 µg/mL; Acris, Herford, Germany). All antibodies were conjugated with Alexa Fluor Protein Labeling Kits (Life Technologies, Grand Island, NY, USA). Alexa Fluor 488 was used for anti-C3c and anti-FH and Alexa Fluor 555 for anti-*Borrelia* antibodies. Samples were washed, and fluorescence was examined with a Nikon Eclipse E600 Confocal Microscope and Nikon Confocal Microscope EZ-C1 2.20 software (Nikon, Chiyoda, Tokyo, Japan).

Analysis of adsorbed proteins was also performed by SDS-polyacrylamide gel electrophoresis under reducing conditions. The proteins were either silver stained (Bio-Rad, Hercules, CA, USA) or transferred to a polyvinylidene fluoride (PVDF) Immune-Blot membrane (Bio-Rad). Detection was performed using biotinylated antibodies against human FH (0.57 µg/ml; Binding Site) and C3c (1.09 µg/ml; Dako), followed by streptavidin-HRP (1∶500; GE Healthcare). Proteins were visualized with ECL Plus Western blot detection system (GE Healthcare). Human C3, C3b, iC3b and FH, purified as described earlier, served as controls [24]. The relative amount of *Borrelia* and total protein was visualized by SDS-PAGE followed by silver staining. A 20 kDa-band of bacterial orgin, most likely OspE [25] was used as loading control in the Western blot experiments [26]. Six separate blots were performed using plasma from different donors. Quantification of the bound proteins was performed using public domain software (Java-based ImageJ software) from the NIH. The β-chain was chosen for quantification of the amount of bound C3-fragments because it is not subject to proteolytic cleavage resulting in transformation to C3b and iC3b.

### Labeling of spirochetes with FITC

Spirochetes were labeled by a modification of the procedure described by Hazenbos *et al*. [27]. *B. garinii* LU59 and *B. afzelii* K78 were washed with 2 mL of 50 mM sodium carbonate with 100 mM sodium chloride, pH 7.4 (Sigma Aldrich), then centrifuged at 4500 *g* for 5 min. The pellets were suspended in 1 mL fluorescein isothiocyanate (0.5 mg/mL; FITC; Sigma Aldrich) in 50 mM sodium carbonate and 100 mM sodium chloride, pH 7.4, and incubated at RT for 20 min on a shaker, washed three times with 4 mL PBSCa^2+^, centrifuged at 4500 *g* for 5 min, and suspended in 2 mL PBSCa^2+^. The spirochetes were visualized by fluorescence microscopy and their motility was unchanged by the labeling procedure [20], [21].

### Phagocytosis of spirochetes

Samples of 50 µL FITC-labeled *B. garinii* LU59 or *B. afzelii* K78 spirochetes (10^8^/mL blood) were incubated in 450 µL hirudin blood without (n = 3) or with (n = 1) *Borrelia*-specific antibodies for 30 min at 37°C in 2-mL tubes. EDTA, which binds the Ca^2+^ and Mg^2+^ that are necessary for a functional complement system and therefore completely inhibits complement activation, was used at a final concentration of 10 mM as a negative control. Two additional inhibitors were used, 50 µM compstatin (Ac-ICV(1-MeW)QDWGAHRCT-NH_2_), a synthetic peptide inhibiting the activation of C3 by the two C3 convertases [28]; and 5.5 µM C5a receptor antagonist (PMX53; C5aRa), which blocks the C5a-mediated up-regulation of granulocytes and monocytes. A scrambled peptide of C5aRa (5.5 µM) was used as a control. The peptides were a kind gift from Dr. John D. Lambris (the University of Pennsylvania, Philadelphia, PA, USA). Reactions were stopped by the addition of 10 mM EDTA (final concentration). Samples (100 µL) of hirudin blood from the incubations were transferred to new tubes, and 2 mL AKC lysis buffer (155 mM NH_4_Cl, 10 mM KHCO_3_, 0.1 M EDTA; Sigma Aldrich) was added to remove and lyse the red blood cells. The reaction was allowed to continue for 5–10 min until the supernatants were clear red. After centrifugation at 400 *g* for 5 min, the supernatants were discarded. The pellets (leukocytes) were washed with 3 mL PBS supplemented with 1% (w/v) bovine serum albumin (BSA; Sigma Aldrich), and then 1.5 mL PBS with 1% paraformaldehyde (Apoteket, Gothenburg, Sweden) and 0.075% trypan blue were added to quench extracellular fluorescence [29], [30]. Granulocytes and monocytes were analyzed according to size (forward scatter) and granularity (side scatter) using flow cytometry (Cell lab Quanta SC, Beckman Coulter, Miami, FL, USA); 5,000 granulocytes were collected, resulting in the collection of ∼500 monocytes. Data are presented as mean fluorescence intensity (MFI). Phagocytosis without complement inhibitors was set to 100%.

### Cytokine and chemokine release

Hirudin blood without anti-*Borrelia* antibodies (n = 3, same individuals as for the phagocytosis experiments described above) was used. Washed spirochetes (10^8^/mL) were incubated in 450 µL of hirudin blood at 37°C for 4 h, with or without the addition of complement inhibitors at a final concentration of 50 µM for compstatin and 100 µg/ml for the monoclonal antibody eculizumab (Soliris, Alexion, Pharmaceuticals, Cheschire, CT, USA) which binds to C5 and prevents its activation. Hirudin blood incubated without spirochetes was used as a control. All incubations were performed in duplicate. Further complement activation was stopped by the addition of 10 mM EDTA (final concentration) and centrifuged at 3000 *g* for 20 min, and the resulting plasma samples were frozen immediately at −80°C. In addition, plasma samples for determination of base-line level of each cytokine or chemokine for each donor were collected directly, without incubation. Complement activation was monitored as the generation of the activation products C3a and sC5b-9 complexes (data not shown). Cytokine and chemokine concentrations were measured by Luminex xMAP technology (Milliplex Human Cytokine/Chemokine Kit, Merck Millipore, Billerica, MA, USA) according to the instructions provided by the manufacturer. The analyzed cytokines and chemokines were: IL-1β, IL-6, IL-10, IL-12(p70), IL-17A, IL-23, TNF, GM-CSF, CXCL10 (IP-10), CXCL1 (GRO-α), CCL22 (MDC), CXCL8 (IL-8), CCL20 (MIP3-α), and CCL2 (MCP-1). Values under the detection limit were given half the value of the lowest point of the standard curve. The inter-assay coefficients of variation (CV) were 3.5–16.4%, and the intra-assay CVs were 3.9–10.5%, according to the manufacturer.

### Statistics

All data were normalized against plasma without bacteria that was treated in the same way as the samples; data are presented as means ± SEM. Statistical analysis of data was performed using Prism version 6.0 for Macintosh software (GraphPad, San Diego, CA, USA). Differences between means were evaluated using paired t-tests and one-way ANOVA followed by Dunnetts post hoc test, and p-values<0.05 were considered to be significant.

## Results

### Susceptibility of *B. garinii* LU59 and *B. afzelii* K78 to complement

*B. garinii* LU59 was highly sensitive to complement with 22 and 40% motile spirochetes remaining after incubation in hirudin plasma diluted 1∶3 respectively 1∶9. No motile spirochetes were found after incubation with undiluted plasma, while the presence of neither heat-inactivated nor native plasma in higher dilutions affected the viability. Complement activation by *B. garinii* LU59 was most likely induced by the alternative pathway since it requires high concentration (>10%) of all participating components [31]. Incubation with hirudin plasma under these conditions did not affect the motility of *B. afzelii K78.*

### Complement activation induced by spirochetes

**Figure 1:**
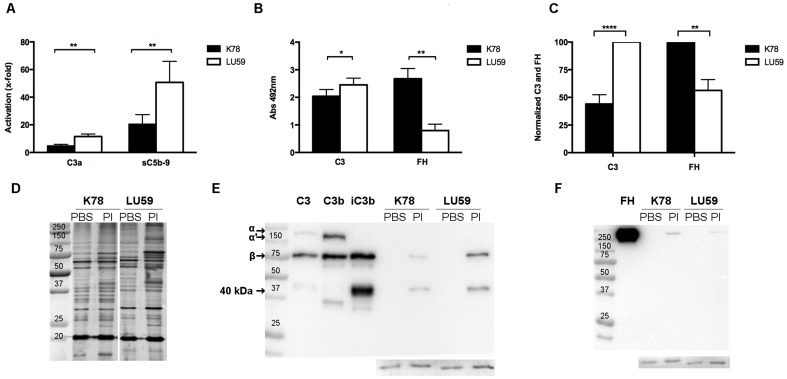
Complement activation and adherence of complement proteins to *Borrelia* spirochete*s* incubated in hirudin plasma. A) The ability of the *Borrelia* strains *B. afzelii* K78 and *B.gariini* LU59, resistant and sensitive to complement activation, respectively, to activate complement in hirudin plasma (n = 8) was assessed by analyzing the generation of C3a and sC5b-9 using ELISA. Data are normalized against plasma incubated without bacteria and are presented as means ± SEM. B) *Borrelia* were incubated with hirudin plasma (n = 6), and bound proteins were analyzed using an in-house-developed particle ELISA. Data are shown as absorbance values of bacteria incubated in plasma, normalized against bacteria incubated in PBSCa^2+^, and are presented as means ± SEM. C) Quantification of bound C3-fragments (using the β-chain that is not proteolytically cleaved) and FH. Results are presented normalized to either K78 or LU59 as mean of six Western blots. D) Silver stained SDS–PAGE of *B. afzelii* K78 and *B.gariini* LU59 non-opsonized (PBS) or opsonised (Pl). In addition, C3 and its fragments (E) and FH (F) bound to bacteria incubated with plasma (Pl) were visualized using Western blot with bacteria incubated in PBSCa^2+^ as controls, and purified C3, C3b and iC3b or FH as references. Insert: loading control, consisting of a 20kDa *Borrelia* protein that was detected on the membranes after detection with antibodies.

Complement activation by spirochetes in hirudin plasma was monitored by the generation of C3a and sC5b-9 complexes. When plasma without *Borrelia*-specific antibodies was used, higher levels of both activation products were found in plasma incubated with *B. garinii* LU59 when compared to plasma incubated with *B. afzelii* K78 (p<0.01) (Figure 1A). An identical experimental setup using plasma with anti-*Borrelia* IgG antibodies (n = 1) showed a higher activation of complement than when the antibodies were not present. In this setting, no significant difference was found between the two bacterial strains (data not shown).

### Quantification of C3/C4 fragments, FH, and C4BP bound to spirochetes

In order to attempt to explain the differences in complement activation between the two strains of bacteria, we analyzed the regulatory proteins FH and C4BP, as well as fragments of C3 and C4 bound to the spirochetes. An ELISA-like assay was developed in which spirochetes were incubated in plasma to allow the binding of complement proteins. *B. garinii* LU59 was found to bind significantly more C3-fragments compared to *B. afzelii* K78 (p p<0.05) while the opposite was true for binding of the regulatory protein FH (p<0.01) (Figure 1B). Similar differences, i.e., that the total amount of C3 bound to LU59 was more than double compared to K78 (p<0.0001), while the opposite binding was true for FH (p<0.01), were found by densitometric scanning of western blots (Figure 1C). In addition, both C4 (fragments) and the inhibitor C4BP were detected, albeit at low levels and with no significant difference between the two strains (data not shown).

In addition, immunostaining was used to ascertain that the detected C3 and FH indeed adhered to the spirochetes (data not shown). Silver staining of SDS-gels showed equal amount of bacteria in each sample and similar amounts of protein for both strains after incubation in plasma (Figure 1D). Western blots were performed to investigate the structure of the bound C3 (i.e. C3, C3b or iC3b), and whether FHL-1 and/or FH related proteins (FHR), bound in addition to FH (as well as for quantification). One representative example of six blots is shown (Figure 1E and F). These experiments were performed with an anti-C3c antibody that also detects degraded fragments of C3, and with an anti-FH antibody with an affinity for the FHL-1 and FHR proteins. Western blots showed that the C3 bound to both strains of spirochetes was quantitatively degraded to its inactive form iC3b, which is unable to form the alternative convertase but still functions as an opsonizing fragment. In the lanes containing opsonized *Borrelia*, the transition to iC3b is visualized by the total degradation of the α-chain and the appearance of the 40 kDa band (Figure 1E).

### Phagocytosis of spirochetes

**Figure 2:**
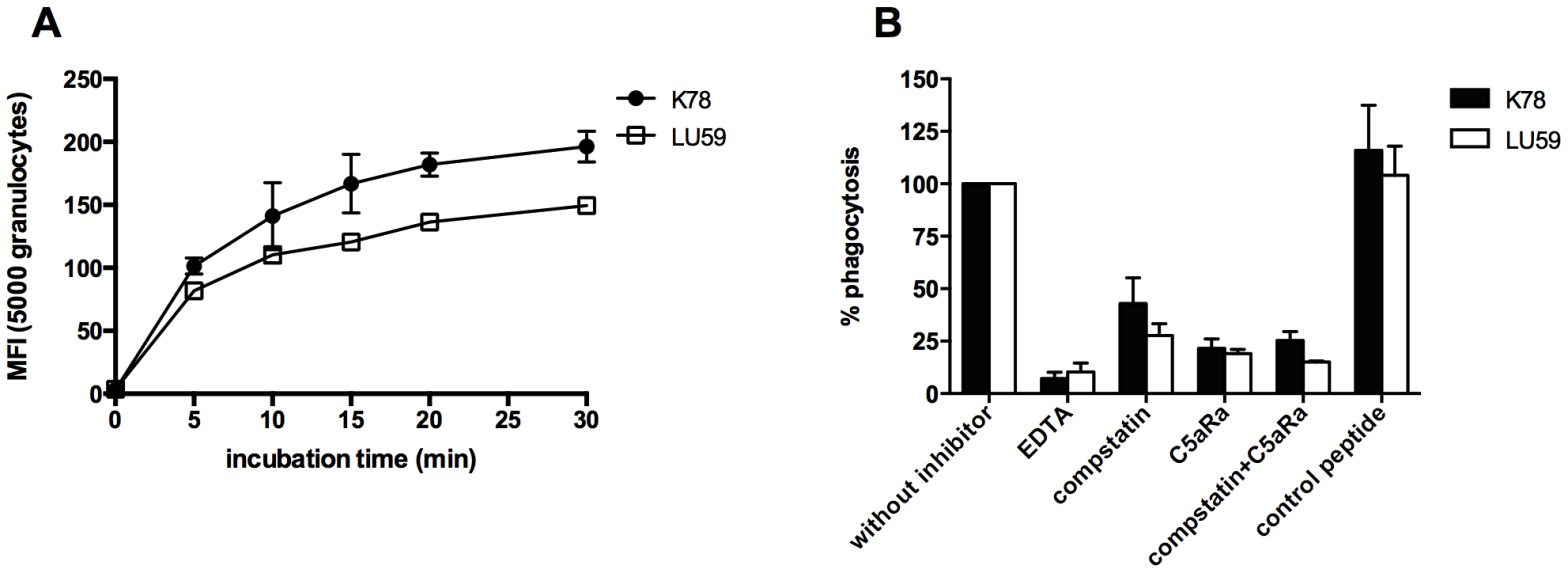
Importance of complement activation for efficient phagocytosis. FITC-labeled *B. afzelii* K78 and *B.garinii* LU59 were incubated in hirudin blood to study phagocytosis by measuring the mean fluorescence intensity (MFI) for granulocytes. A) Phagocytosis of each bacterial strain was monitored for 30 min. Results are presented as mean ± SEM of two observations. B) The importance of complement activation in phagocytosis was analyzed by incubating *Borrelia* spirochetes in hirudin blood (n = 3) with the addition of complement inhibitors acting at different stages in the activation cascade. Compstatin selectively inhibits the proteolytic activation of C3 and a C5a receptor antagonist (C5aRa) that blocks the C5a-mediated up-regulation of granulocytes and monocytes. A scrambled peptide of C5aRa was used as a control. The mean fluorescence intensity (MFI) for each bacterial strain incubated in hirudin blood without complement inhibitors was set to 100%. EDTA: ethylene diaminetetraacetic acid. C5aRa: C5a receptor antagonist.

Phagocytosis of *B. afzelii* K78 by granulocytes was more efficient than that of *B. garinii* LU59 within a 5- to 30-min time period, (Figure 2A) with significant difference (p<0.05) at 30 min). To elucidate the importance of complement activation for efficient phagocytosis, we used inhibitors acting on different levels of the complement cascade (Figure 2B). The C5aRa showed an approximately 80% inhibition for both strains, with no synergy between the two inhibitors. The decrease in phagocytosis of each *Borrelia* strain by the two inhibitors, alone or in combination, were highly significant (p<0.0001). Similar results were obtained regarding phagocytosis by monocytes (data not shown). In the presence of *Borrelia*-specific antibodies, phagocytosis of *B. garinii* LU59 was inhibited by 70% in the presence of either inhibitor. *B. afzelii* K78 was 45% inhibited by compstatin and 60% by C5aRa (data not shown).

### Cytokine and chemokine release

**Figure 3:**
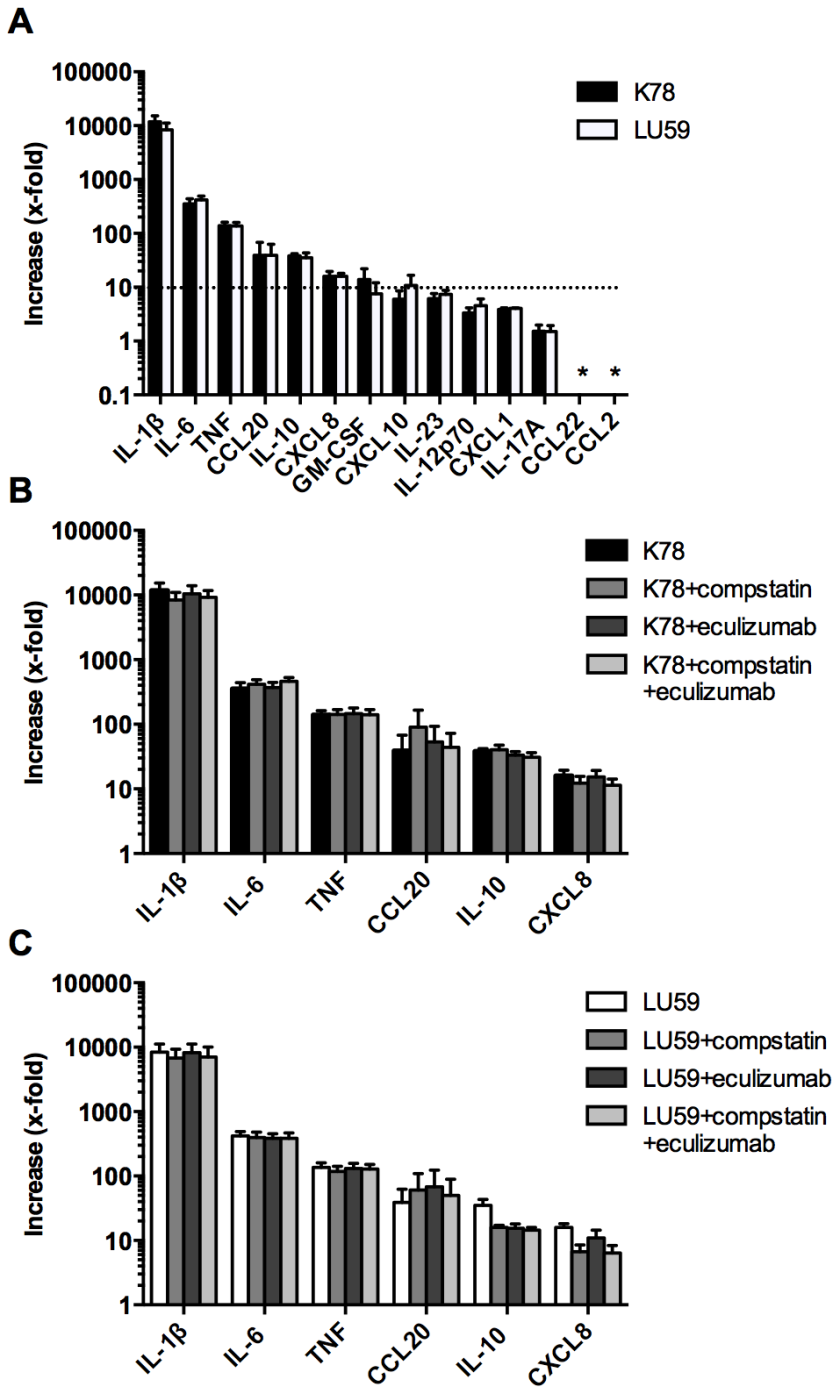
Cytokine and chemokine levels in plasma as a response to incubation with *Borrelia* spirochetes. Spirochetes were incubated in whole blood (n = 3) in duplicate for 4 h, and the cytokine/chemokine production was then measured using a Luminex-based assay. Results are normalized against the values for plasma without the addition of bacteria. (A) The x-fold increase in the panel of cytokines and chemokines analyzed in this report showed no difference between the complement-resistant *B. afzelii* K78 and -sensitive *B. garinii* LU59. (B and C) The importance of complement activation was further investigated using two complement inhibitors: compstatin, which selectively inhibits the proteolytic activation of C3, and eculizumab, an anti-C5 antibody blocking the cleavage of C5 and therefore further complement activation and C5a mediated signaling. Data are presented as means ± SEM.

As a measurement of the early inflammatory response to *Borrelia* infection, we analyzed the levels of secreted cytokines and chemokines after 4 h of incubation of hirudin blood with or without *Borrelia* spirochetes. After exposure to *Borrelia* spirochetes, we saw increases in the secreted levels of IL-1β (10,000-fold), IL-6 (400-fold), TNF (140-fold), CCL20 (40-fold), IL-10 (35-fold), CXCL8 (15-fold), GM-CSF (10-fold), CXCL10 (8-fold), IL-23 (7-fold), IL-12p70 (4-fold), CXCL1 (4-fold), and IL-17A (1.5-fold), although the levels did not differ significantly between *B. afzelii* K78 and *B. garinii* LU59 (Figure 3A). CCL22 and CXCL1 did not reach levels above the detection limit of the assay. The pro-inflammatory cytokines IL-1β, IL-6, TNF, CCL20, and CXCL8, together with the anti-inflammatory IL-10, increased the most, i.e., more than 10-fold in comparison with the control. Therefore, these cytokines and chemokines were used to further elucidate the importance of complement activation for their release (Figure 3B and C). Inhibition of complement at the levels of C3a and C5a generation showed no significant effect on cytokine or chemokine release, the only exception being CXCL8, which showed a significant 60% decrease for LU59 in the presence of compstatin (Figure 3C). The background levels of all cytokines (i.e. in plasma which was collected immediately, without incubation) were all below the detection limit in each blood donor.

## Discussion

In the present study, we have investigated functional aspects of the early innate immune response to *B. afzelii* K78 and *B. garinii* LU59 by examining complement activation, phagocytosis, and cytokine and chemokine release.

One main finding regarding complement activation was the significant difference in found between the two strains of *Borrelia*. In accordance with previous findings [32], we found that the complement-sensitive strain *B. garinii* LU59 induced a higher level of complement activation, as measured by the generation of C3a and sC5b-9 in plasma and detected by ELISA. Complement activation induced by the presence of *Borrelia*-specific antibodies resulted in a significant increase in generated sC5b-9, in agreement with other studies of the deposition of C5b-9 on the bacterial surface [8], [18], [33], [34]. Moreover, although they were measured in only one case (because we identified only one donor with anti-*Borrelia*-specific antibodies, despite having screened a large number of blood donors) the presence of *Borrelia*-specific antibodies compensated for the differences in complement activation between the sensitive and the resistant strain, thus indicating the benefit of pre-existing antibodies for efficient clearance.

It has previously been shown that all three pathways of complement activation are involved in complement-mediated killing of *Borrelia* spirochetes, although the alternative pathway is believed to be the most important [12]. Recruitment of complement regulators to the bacterial surface is an established mechanism used by pathogens to avoid complement attack [35]–[37]. Here we investigated the two main regulators of the alternative and classical pathway, FH and C4BP and both of which act as co-factors for factor I (FI)-mediated degradation of C3b and C4b, respectively [38]–[40]. Regulatory proteins bound to the membrane of *B. garinii* LU59 and *B. afzelii* K78 were quantified by an in-house-developed particle ELISA, as well as by western blotting (FH) confirming that *B. afzelii* K78 had recruited more FH (at least twice as much) than had the complement-sensitive *B. garinii* LU59, an observation also made by others [10], [13], [41]. Furthermore, we detected significantly higher amounts of deposited C3 on LU59 by both techniques, more than twice the amount found on K78, which is in agreement with the higher amount of C3a (and subsequently sC5b-9), generated by LU59. Interestingly, western blot analysis showed that the deposited C3 had been quantitatively degraded to iC3b on both strains despite the fourfold difference in C3/FH ratio, suggesting that FH may not be the limiting factor under the experimental conditions used here.

In addition, low amounts of C4BP were detected on both strains, with a non-significantly higher level on *B. garinii* LU59. Binding of C4BP to *B. garinii* in the literature is contradictory, studies in which no binding of C4BP was detected when a similar experimental setup has been reported [13], [32] as well as studies in line with data presented here where binding of C4BP is observed [15].

Microbes are generally cleared from the body by phagocytosis. The *Borrelia* spirochetes used in this study were phagocytosed by both granulocytes and monocytes, a finding that has previously been demonstrated using other *B. burgdorferi* s.l [42]–[44]. For these studies we used FITC-labeling, which is a standard technique to study opsonization and phagocytosis of pathogens [45], [46]. Intriguingly, the phagocytosis assay showed that *B. afzelii* K78 was more extensively phagocytosed than was *B. garinii* LU59 at all time points, a result that has not been reported previously. It is most unlikely that this difference in phagocytosis is a consequence of differences in viability since live and dead bacteria are phagocytosed by granulocytes at similar rate [47].

When we examined the importance of complement activation in phagocytosis by using several complement inhibitors interfering with the activation of complement at different stages in the activation cascade, we found that phagocytosis was obliterated in the presence of the complement inhibitor EDTA, a chelator that binds Ca^2+^ and Mg^2+^. These ions are necessary for a functional complement system but also for a number of other biochemical reactions so the addition of EDTA could possibly have affected other mechanisms involved in phagocytosis, and not uniquely the complement system. Therefore, we also tested the effects of complement inhibitors acting at the levels of C3 and C5, respectively. Both inhibitors produced a 50–80% reduction in phagocytosis, without any obvious synergistic effects. Although it can not be totally excluded that the FITC-labeling procedure *per se* may affect activation and binding of complement proteins to the pathogen surface, the marked effect on phagocytosis seen using compstatin and C5aRa makes this option less likely. The effect of complement inhibition on phagocytosis was less prominent in the presence of *Borrelia*-specific antibodies. Consequently, complement activation is most important but not completely essential for phagocytosis. This observation is supported by other *in vitro* studies of human neutrophils as well as by *in vivo* studies using C3 knockout mice [42], [48].

Regarding the analysis of early cytokine and chemokine release in human blood in response to live spirochetes, we found a rapid and pronounced pro-inflammatory response, with high levels of major inflammatory mediators such as IL-1β, IL-6, TNF, and CXCL8, an observation that is in line with previous reports [19], [49], [50]. Interestingly, the T helper (Th) 17-recruiting chemokine CCL20 was also highly induced, as well as the Th17-inducing cytokine IL-23. In addition, pro-inflammatory IL-6 aids in Th17 induction, and CXCL8 is in part associated with Th17. These findings are in line with recent observations of Th17-like immunity in neuroborreliosis [51]–[53] and Lyme arthritis [54]. Furthermore, Th1-inducing IL-12p70, as well as Th1-recruiting CXCL10, were among the highly induced cytokines, whereas the Th2-recruiting CCL22 was not induced at all. Thus, the pattern we observed could reflect the ensuing adaptive response that is characterized by Th1 predominance over Th2 reactivity [51], [53], [55]–[57]. Notably, we found that already after 4 h of incubation with *Borrelia* spirochetes, there were significantly elevated levels of IL-10. IL-10 is considered a key negative regulator of pro-inflammatory cytokine release and function [58]. It has been shown that *B. burgdorferi* s.l. induces the secretion of IL-10 in mononuclear cells, and the clearance of *B. burgdorferi* s.l. in IL-10-deficient mice is 10-fold higher than that in wild-type mice [59]. These studies, together with our findings suggest, that *B. burgdorferi* s.l. itself may induce IL-10 to inhibit the host defense.

Activation of pattern recognition receptors (PRR), such as toll-like receptors (TLR), by conserved lipoproteins on the spirochetal membrane enhances phagocytosis and antigen presentation, as well as triggering the production and release of inflammatory cytokines [60], [61]. TLR activation induced by *Borrelia* spirochetes has been shown to induce the activation of the transcription factor NFκβ, which is involved in the production of pro-inflammatory cytokines [62], [63]. In the present study, we showed a 10- to 10,000-fold increase in the release of pro-inflammatory cytokines when compared to background levels. IL-1β increased the most (i.e., 10 times more than IL-6). IL-1β has previously been connected with the activation of PRR as a response to *Borrelia* spirochetes [64]. It has recently been suggested that autophagy is responsible for an increased production of IL-1β and IL-6 in peripheral mononuclear blood cells as a response to *B. burgdorferi* s.l. [65]. Furthermore, differences in cytokine response has been described following phagocytosis depending on the viability of E.coli: both dead and live bacteria are reported to induce, e.g. IL-6 to similar degree, while generation of IL-1β is much reduced after phagocytosis of dead bacteria. This difference has been attributed to the intracellular release of bacterial mRNA from live, ingested bacteria, which leads to inflammasome activation which is required for processing of pro-IL-1β to mature IL-1β [66].

Our results showed that inhibition of complement activation decreased phagocytosis without affecting the release of any of the detected cytokines and chemokines, except for CXCL8, indicating that phagocytosis is not crucial for the pro-inflammatory response. This finding is in contrast to other observations that have shown a significant decrease in pro-inflammatory cytokines when phagocytosis or phagosomal signalling is blocked [43], [67]. However, these studies were made using systems of purified monocytes, without the presence of active serum, so the impact of complement activation could not be evaluated.

Based on our findings, we propose that the complement-sensitive strain *B. garinii* LU59, which is less prone to inhibit the alternative pathway by recruiting FH, would seek an immunologically privileged site such as the central nervous system (CNS). This behavior would constitute an optimal strategy for escaping the effects of complement in early infection and could at least in part explain the neurotropism observed in this genospecies [6]. However, it cannot be the full explanation, since *B. garinii* OspA serotype 4, which has been repeatedly isolated from human CSF [68], is able to bind FHL-1 quite efficiently [69]. During established CNS borreliosis, we have previously demonstrated evidence of complement activation via the classical pathway in CSF [70]. However, *B. garinii* has previously been shown to be able to bind C4BP [15], a finding that is consistent with the *in vitro* experiments performed here. Therefore, C4BP could constitute an important virulence factor for spirochetal survival in the presence of antibodies.

Taken together, our findings suggest that the early innate immune response, including complement activation, phagocytosis, and the release of cytokines and chemokines, may influence the course of *Borrelia* infection. We have shown: 1) that the complement-resistant strain *B. afzelii* K78 recruits more FH and hence induces a lower activation of complement than does the complement-sensitive *B. garinii* LU59, a finding that in part could explain the neurotropism of the sensitive strain; 2) that both granulocytes and monocytes participate in the phagocytosis of *Borrelia* spirochetes and that complement activation is extremely important, but not crucial, for spirochetal phagocytosis; 3) that the early cytokine/chemokine response to *Borrelia* spirochetes was dominated by pro-inflammatory mediators and also by inducers and recruiters of Th1- and Th17-like immunity; 4) that the assessed cytokine/chemokine response was not affected by the complement system in this *in vitro* model. Since only minor differences were found in response to the two strains, a valid conclusion from this work could be that there is enough redundancy in complement sufficient subjects to eradicate *Borrelia* infections.
